# Avian Orthoavulavirus Type-1 as Vaccine Vector against Respiratory Viral Pathogens in Animal and Human

**DOI:** 10.3390/vaccines10020259

**Published:** 2022-02-08

**Authors:** Julianne Vilela, Mohammed A. Rohaim, Muhammad Munir

**Affiliations:** 1 Division of Biomedical and Life Sciences, Lancaster University, Lancaster LA1 4YG, UK; j.vilela@lancaster.ac.uk (J.V.); m.a.rohaim@lancaster.ac.uk (M.A.R.); 2 Department of Virology, Faculty of Veterinary Medicine, Cairo University, Giza 12211, Egypt

**Keywords:** avian orthoavulaviruses type-1, human, animal, vaccines, mitigations strategies

## Abstract

Avian orthoavulaviruses type-1 (AOaV-1) have recently transitioned from animal vaccine vector to a bona fide vaccine delivery vehicle in human. Owing to induction of robust innate and adaptive immune responses in mucus membranes in both birds and mammals, AOaVs offer an attractive vaccine against respiratory pathogens. The unique features of AOaVs include over 50 years of safety profile, stable expression of foreign genes, high infectivity rates in avian and mammalian hosts, broad host spectrum, limited possibility of recombination and lack of pre-existing immunity in humans. Additionally, AOaVs vectors allow the production of economical and high quantities of vaccine antigen in chicken embryonated eggs and several GMP-grade mammalian cell lines. In this review, we describe the biology of AOaVs and define protocols to manipulate AOaVs genomes in effectively designing vaccine vectors. We highlighted the potential and established portfolio of AOaV-based vaccines for multiple respiratory and non-respiratory viruses of veterinary and medical importance. We comment on the limitations of AOaV-based vaccines and propose mitigations strategies. The exploitation of AOaVs vectors is expanding at an exciting pace; thus, we have limited the scope to their use as vaccines against viral pathogens in both animals and humans.

## 1. Introduction

Emerging and re-emerging pathogens have recently made a significant impact on global economies and worldwide public health [1]. Several novel and re-emerging viral infections in humans and animals have been documented in recent decades, including Marburg virus, Lassa fever, Ebola, HIV/AIDS, influenza, Hanta, MERS [2,3], and, most recently, the global pandemic caused by SARS-CoV-2 (COVID-19) [4]. Vaccination has proven to be the most effective means of protection against emerging and re-emerging pathogens. With advancements in immunology, molecular biology, and microbiology, vaccine development technology is continuously advancing [5]. Recombinant viral vectors, in particular, represent a strong and promising platform to produce safe, immunogenic and effective vaccines without the cultivation and treatment of live pathogens, particularly those, which are lethal to human and animals. Through these advances, recombinant viral vectors-based vaccines are now a safer, yet powerful tool for developing vaccines and are able to induce long term immunity [6,7].

Herpesvirus, adenovirus, and vaccinia virus (DNA viruses) have genomes that encode a huge number of proteins and were initially utilized as vaccines or vectors in gene therapy [8,9,10]. On the other hand, RNA viruses have been evaluated as foreign immunogen delivery vehicles since the advent of reverse genetic systems [11]. The demand for efficient vaccines has increased dramatically in order to combat emerging and re-emerging infectious diseases and overcome the drawbacks of traditional vaccines (live attenuated and inactivated) [11]. Live attenuated vaccines have historically provided the most effective protection against viral infection [12] compared to inactivated vaccines that were ineffective and require a high containment laboratory for handling of highly pathogenic pathogens and the risk of incomplete inactivation. In addition, there are safety concerns about the risk of pathogen reversion to the wild-type phenotype, as seen with some traditional live attenuated vaccines beside a failure to develop live attenuated vaccines for many important pathogens.

Viral vector-based vaccines, which rely on the delivery of a single or more unrelated, modified virus antigens, are a highly versatile platform with many advantages over established vaccine technology. Either live (replicative but often damped) or non-replicating vectors are used by this technology [13]. In general, replicating viral vector vaccines have the characteristics of a live vaccine without the need for the whole pathogen to be involved or for the pathogen to be cultivated along with their ability to deliver the foreign antigen intracellularly, inducing humoral, cellular, and mucosal immune responses [14]. Specifically, vectored vaccines carry multiple advantages and aspects for usage; zoonotic viruses that have safety problems (i.e., SARS-CoV-2), viruses that lose infectivity because of physical instability, i.e., respiratory syncytial virus (RSV), and viruses that may exchange genes with other circulating viruses (i.e., coronaviruses and influenza viruses). Therefore, vectored vaccines can be used for vaccine development against highly pathogenic viruses and do not necessitate the use of biosafety containment laboratories at a higher level [15].

Avian orthoavulavirus type-1 (AOaV-1), previously known as Newcastle disease virus (NDV), is a non-segmented negative-sense RNA virus belonging to paramyxovirus, which naturally infects birds, causing severe economic losses globally [16]. Although AOaV-1 is naturally monotypic, antigenic and genetic diversity have been reported among AOaV-1 isolates. Two distinct classification methods for AOaV-1 are now in use worldwide. When comparing the isolated sequences, one of the systems separates AOaV-1 into two primary divisions, Class I and Class II. Class I is further subdivided into nine genotypes while class II is subdivided into ten genotypes. Class II viruses have been studied in detail, and the early 1930–1960 genotypes I, II, III, IV, and IX have 15,186 nucleotides. [17,18,19]. Genotypes V, VI, VII, VIII, and X that appeared late (after 1960) comprise 15,192 nucleotides.

**Figure 1:**
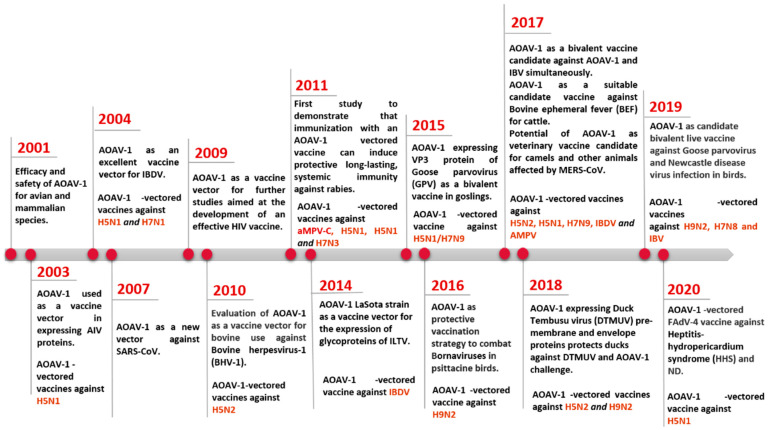
Progress on the use of AOaV-1 as viral vector for vaccine development.

Currently, live vaccines based on lentogenic AOaV-1 strains Hitchner B1, LaSota, Fuller (F), and mesogenic strain R2B are commonly utilised. AOaV-1 strain F is a low-virulence virus that has been investigated in different countries throughout Europe, Africa, and Asia in the form of a live vaccine [20]. AOaV-1 research is not only focused on its pathobiology, but also on its potential use as a vaccine vector to develop novel vaccines for poultry and mammals, including humans [15,21,22]. Numerous AOaV-1-vectored vaccines expressing protective antigens from various pathogens have been developed since the first recombinant AOaV-1 expressing a foreign gene in 2000 (Figure 1) [23]. In addition, AOaV-1 encodes six major proteins, resulting in less immune response competition between vector proteins and the expressed foreign antigen. The AOaV-1 replicates in the cytoplasm do not integrate into the host cellular DNA, and do not cause persistent infection. In addition, the AOaV-1-vectored vaccine can also be used as a “differentiating infected from vaccinated animals” (DIVA) vaccine. Therefore, the aim of this review is to summarise the recent advances in the field of the AOaV-1 vectored vaccine and discuss its potential application against emerging and re-emerging animal and human viral pathogens.

## 2. Genomic and Biological Features of Avian Orthoavulavirus Type-1

**Figure 2:**
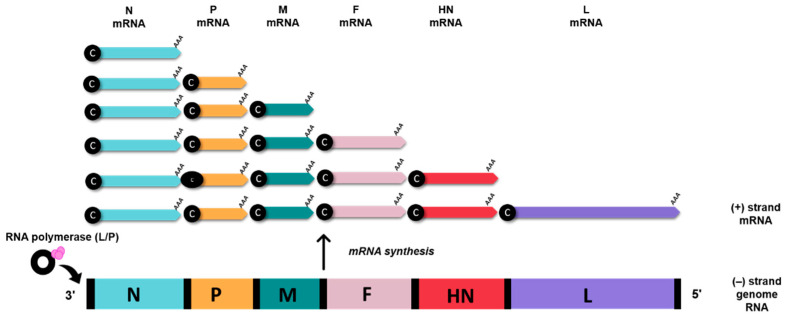
AOaV-1 mRNA synthesis. Viral (-) strand genome serves as templates for the generation of sub-genomic mRNAs. The RNA polymerase (L/P) initiates mRNA synthesis at the beginning of the N gene, near the 3′ end of the viral genome. Small black spheres denote 5’ cap (c), AAA denotes poly (A) tail.

The AOaV-1 causes one of the most significant avian viral diseases, responsible for severe economic losses globally [1]. AOaV-1 is taxonomically classified a member of the order *Mononegavirales*, family *Paramyxoviridae*, sub-family *Paramoxivirinae* under genus *Avulavirus* [1,2]. The AOaV-1 virion structure is pleomorphic but mostly roughly spherical with diameters of around 100–500 nm [2,3]. The AOaV-1 genome is around 15 kb of linear single-stranded, negative-sense RNA with six essential genes from 3′ to 5′ direction- nucleocapsid (N), phosphoprotein (P), matrix protein (M), fusion protein (F), haemagglutinin-neuraminidase protein (HN), and large polymerase protein (L) [24]. Each gene is identified by the presence of signal sequences at the start (GS—gene-start) and at the end of the gene (GE—gene-end). Additionally, the presence of intergenic sequences (IGS) separates the genes from each other [2]. All AOaV-1 genes code for a single major protein; however, due to transcriptional editing or differential initiation of gene P, mRNA may result in the formation of a non-structural V and/or W protein [24]. The viral RNA polymerase (L protein) initiates the transcription of the viral genome at the 3′ terminal nucleotide, sequentially terminating and reinitiating at multiple start/stop gene sites. At the end of each gene, a fraction of RNA polymerase dissociates from the genome and is unable to restart transcription to the next downstream gene, thus resulting in diminished quantities of mRNAs for genes located further the 3′ end of the genome. This mechanism enables the balance in synthesising proteins that are needed in high concentration such as N and M and proteins needed in smaller amounts (i.e., HN and L) (Figure 2) [24].

Virulence and infectivity of AOaV-1 depends on both F and HN proteins [4,5]. Co-expression and interaction of homologous F and HN proteins are required for virus fusion. The HN protein is involved in virus-specific membrane fusion. Through haemagglutination and neuraminidase activities, the HN protein facilitates the interaction between host receptors and virus, which allows the host membrane and viral membrane to be closer together, granting the F protein to come in contact with the host cell, so the virus will be able to penetrate the cell surface [3]. The F protein mediates the fusion of the AOaV-1 with the host cell that undergoes a conformational change, allowing the cleavage of the protein precursor F_0_ to F1/F2 subunits, which also brings the viral envelope closer to the cell membrane, leading to the fusion of the two membranes [24]. Thus, the amino acid sequence of the cleavage site of the F protein is theorised to be a major determinant of infection [2,4,6]. 

## 3. AOaV-1 as a Viral Vaccine Vector

**Figure 3:**
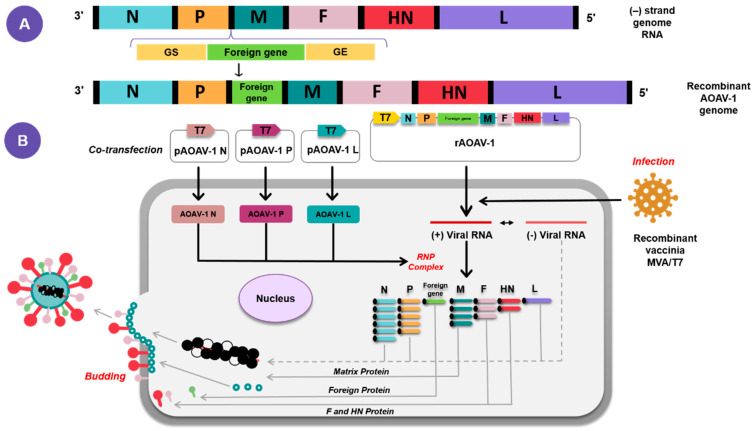
Diagrammatic representation of the reverse genetics strategy for AOAV-1 rescue. Construction of recombinant AOAV-1 containing foreign gene (**A**). The recombinant AOAV-1 will be co-transfected together with expression helper plasmids encoding the N, P, and L proteins of AOAV-1 and infected with recombinant vaccinia MVA/T7 expressing T7 RNA polymerase (**B**).

Recent advancements in recombinant DNA technologies, molecular biology, and biotechnology provided the platform to further understand AOaV-1 as a viral pathogen, as well as its potential to serve as vaccine and or vaccine vector for humans and animals. Due to the modular nature of transcription, the low recombination frequency, and the absence of DNA phase during replication, AOaV-1 is considered a promising candidate for the development of live attenuated vaccines and vaccine vectors [2]. Using reverse genetics, AOaV-1 can be easily engineered to bear the desired antigens as additional proteins, by using a recombinant viruses recovered from cloned cDNA (Figure 3) [7,8,9,10]. The benefits of AOaV-1 as a viral vector include (1) its ability to replicate efficiently in vivo with high titers; (2) its ability to elicit humoral and cellular immune cell responses; (3) its ease of genome manipulation; (4) its independent replication and failure to integrate into the host genome; and (5) stable expression of foreign proteins [2,6,9]. 

The transfection of cultured cells with plasmids encoding the viral components of functional nucleocapsid, full-length antigenomic RNA, major proteins involved in viral replication and transcription (N, P and L) under the control of bacteriophage T7 RNA polymerase promoter, allows infectious AOaV-1 to be recovered entirely from cloned cDNA, which is also known as reverse genetic technique (Figure 3) [6,7,11,12,13,14]. A foreign gene flanked by AOaV-1 GS and GE sequences was inserted within a non-coding region of the AOaV-1 genome. Foreign genes are more efficiently expressed when they are inserted closer to the 3′ end of the genome due to polar gradient transcription. While it is possible to insert a foreign gene between any two AOaV-1 genes, i.e., the insertion site between the P and M genes has proved ideal for efficient expression of the extraneous proteins [14,15,16,17]. The AOaV-1 adapts the external genes with good stability and can express up to three foreign genes within a single AOaV-1 vector [6].

LaSota and B1 lentogenic AOaV-1 strains were commonly used as vaccine vectors due to their safety records. Mesogenic and velogenic AOaV-1 strains are not being used in chickens as vaccine vector due to their virulence in chickens. An in vivo study for a AOaV-1 vectored vaccine in non-human primates based on the mesogenic strain (Beaudette C) revealed its efficient replication with a substantially higher level of antibodies compared to the LaSota strain vectored vaccine [18], indicating that it would be an efficient vaccine vector.

## 4. Current Application of AOaV-1 as Vaccine Vector for Poultry and Animal Viruses

Efforts for studying the AOaV-1 as a viral vector have given rise to various vaccine candidates expressing antigens of various viral pathogens in poultry, animals, and humans [19]. Preclinical and clinical studies have been conducted to assess the safety, immunogenicity, and protective efficacy of these AOaV-1-vectored vaccines.

### 4.1. Recombinant AOaV-1 Based Vaccines for Poultry

Vaccination with attenuated or killed vaccines has been the primary means of prevention and control of Newcastle disease (ND) globally. Recombinant AOaV-1 vaccines have proven to elicit protective immunity, be highly immunogenic, and can be administered in large scale through drinking water, eye drops, sprays, and even in ovo injection [20]. AOaV-1 vaccines can be produced in a highly cost-efficient manner, as they can be propagated in high quantity in embryonated chicken eggs and even in cell culture. AOaV-1 vaccine strains are appealing as viral vectors for the development of multivalent vaccines against emerging and re-emerging viral pathogens that threaten the poultry industry (Table 1) [9]. The efficacy and success of AOaV-1 as a viral vaccine vector is highly dependent on the strains’ backbone (i.e., naturally attenuated AOaV-1 strains such as LaSota or Hitchner B1, or genetically modified strains with mutations for attenuation) can be used [19,21,22,23]. 

#### 4.1.1. Avian Influenza Virus (AIV)

Among the most important pathogen of poultry and wild birds is the highly pathogenic avian influenza virus (HPAIV), which not only causes major economic losses globally, but also poses a continuous threat to human health [20,25]. In the effort to protect the poultry industry against HPAIV, various AOaV-1 vector-based vaccines were developed by insertion of different hemagglutinin (HA) genes into the AOaV-1 genome (i.e., H5, H6, H7, H9) [26,27,28]. Numerous studies have shown that AOaV-1-vectored influenza vaccines based on the LaSota strain are safe and effective when challenged with HPAI or low pathogenic avian influenza (LPAI) viruses [29,30,31,32,33,34]. Decades of successful usage of AOaV-1 vector as a bivalent vaccine against AIV and AOaV-1 has paved the way for the development of other multivalent vaccines against other economically important poultry pathogens.

**Table 1:** Candidate AOaV-1-vectored vaccines for poultry use.

Pathogen	AOaV-1 Backbone	Antigen	Insert site	Animal Model	Vaccination (Route)	References
H1N1	Hitchner B1	HA	P/M	Mouse	i.v. or i.p.	[16]
H5N1	La Sota	HA	P/M	Chicken/Mouse	o.n. (chicken); i.p (mouse]	[35]
H5N1	La Sota	HA HA + AOaV-1 F	P/M	Chicken	o.n.	[29]
H5N2	La Sota	HA + AOaV-1 F	P/M	Chicken	i.m./spray	[34]
H5N1/ H7N9	La Sota	HA + AOaV-1 F	P/M	Chicken	i.m. or o.n.	[31]
H5N2	La Sota/ Chimeric AOaV-1	HA and NA	HA- P/M NA- M/F	Chicken	i.n	[36]
H5N1	Chimeric AOaV-1 based on AOaV-1 clone 30	HA	F/HN	Chicken	o.n.	[37]
H5N1	Chimeric AOaV-1 strain BC	HA	N/P or P/M	Chicken	o.n.	[25]
H5N1	La Sota/ Chimeric AOaV-1	HA HA and NA HA and M1 HA and NS1	HA- P/M NA, MI, NS1- M/F	Chicken	o.n.	[38]
H5N1	TS09-C	HA/HA1	P/M	Chicken	i.n/i.o.	[39]
H5N1	La Sota	HA	P/M	Duck	i.o	[40]
H5N2	La Sota	HA	P/M	Chicken	i.o.	[41]
H5N1	Beaudette C strain	HA	P/M	Monkey	i.n/i.t.	[22]
H9N2	La Sota	HA HA + AOaV-1 F	P/M	Chicken	o.n/i.m.	[32]
H9N2	rmNA-1 strain	HA HA + AOaV-1 F	P/M	Chicken	o.n.	[42]
H9N2	Chimeric AOaV-1	HA	P/M	Chicken	o.n.	[43]
H7N2	Hitchner B1	HA + AOaV-1 F	P/M	Chicken	i.o.	[28]
H7N2	Hitchner B1	HA	P/M	Chicken	i.o.	[44]
H7N1	Clone 30	HA	F/HN	Chicken	i.n.	[45]
H7N9	LX	HA HA + AOaV-1 F	P/M	Chicken	i.n.	[46]
H7N3	La Sota	HA	P/M	Mouse	i.n.	[26]
H7N8	La Sota/ Chimeric AOaV-1	HA HA and NA	HA- P/M NA- M/F	Chicken	i.n.	[47]
H6N2	Clone 30	HA	F/HN	Chicken/Turkey	o.n.	[48]
IBDV	La Sota	VP2	Upstream of the NP	Chicken	i.o.	[49]
IBDV	F strain	VP2	P/M	Chicken	i.n.	[50]
IBDV	rLaC30L [La Sota; Clone 30]	VP2	P/M	Chicken embryo	in ovo	[51]
ILTV	La Sota	gB or gD	P/M	Chicken	i.n./i.o.	[52]
ILTV	La Sota	gB or gC or gD	P/M	Chicken	o.n.	[53]
IBV	La Sota	S	P/M	Chicken	o.n.	[54]
IBV	La Sota	S1	P/M	Chicken	o.n.	[55]
IBV	La Sota	S1 [multi-epitope]	P/M	Chicken	o.n.	[21]
AMPV	La Sota	G	F/HN	Turkey	i.n./i.o.	[56]
AMPV	La Sota	G + F	F/HN	Turkey	i.n./i.o.	[57]
FAdV	La Sota	fiber 2	P/M	Chicken	i.m.	[58]
GoAstV	SH12	Cap	P/M	Gosling	o.n.	[59]
GPV	NA-1 strain	VP3	P/M	Gosling	s.c.	[60]
DTMUV	GM	prM + E	P/M	Duck	s.c.	[61]
Bornavirus	Clone 30	N or P	F/HN	Cockatiel/Canary	i.m.	[62]

Avian orthoavulavirus type-1 (AOaV-1); Avian influenza virus (AIV); Infectious bursal disease virus (IBDV); Infectious laryngotracheitis virus (ILTV); Infectious bronchitis virus (IBV); Avian metapneumovirus (AMPV); fowl adenovirus (FAdV); Goose origin avastrovirus (GoAstV); Goose parvovirus (GPV); Duck tembusu virus (DTMUV); Hemagglutinin (HA/H/HN); hemagglutinin subunit 1 (HA1); AOaV-1 Fusion (F) protein: transmembrane and cytoplasmic tail of the AOaV-1 F protein; Nucleoprotein (NP); Neuraminidase (NA); Matrix 1 (M1); Nonstructural 1 (NS1); Viral protein 2 (VP2); Viral protein 3 (VP3); glycoprotein (G); glycoprotein B (gB); glycoprotein C (gC); glycoprotein D (gD); spike (S); spike subunit 1 (S1); fiber proteins (fiber 2); Capsid protein (Cap); nucleoprotein (N); phosphoprotein (P); pre-membrane protein (prM); envelop protein (E); intravenous (i.v.); intraperitoneal (i.p.); oculonasal (o.n.); intramuscular (i.m.); intranasal (i.n.); intraocular (i.o.); intratumoral (i.t.); subcutaneous (s.c.).

#### 4.1.2. Infectious Bronchitis Virus (IBV)

Infectious Bronchitis (IB) is a highly contagious viral respiratory disease of poultry causing significant economic losses to the poultry industry worldwide [63]. Control of IB is primarily achieved with live attenuated and inactivated vaccines that can elicit effective humoral and cellular immunity; however, their preparations are time consuming, expensive and complicated [54]. Recently, vectored vaccines based on the LaSota and other AOaV-1 strains have been used to express the spike (S) protein of infectious bronchitis virus (IBV) [21,54,55,64]. Zhao et al. [55] have demonstrated the successful expression and incorporation of a chimeric S1 expression cassette into the AOaV-1 genome, generating a recombinant bivalent AOaV-1 vaccine that can induce an antibody response to IBV following a single dose vaccination. Similar studies were conducted to compare the level of protection of the recombinant AOaV-1 expressing different S proteins (i.e., S, S1 subunit and S2 subunit). [64] revealed that S protein, which contains both S1 and S2 proteins, is the most effective IBV antigen for generation of recombinant AOaV-1 vaccine [64]. In addition, Abozeid et al. [54] reported that single-dose vaccination with recombinant AOaV-1 expressing S protein provided complete protection against IBV and virulent AOaV-1; however, it did not show reduction in tracheal viral shedding. In contrast, Tan et al. [21] have found that recombinant AOaV-1 vaccine expressing multi-epitope cassette derived from S1 subunit of IBV can protect 90–100% chicks with a single-dose vaccination without virus shedding [21]. 

#### 4.1.3. Infectious Bursal Disease Virus (IBDV)

AOaV-1 strain LaSota was used to express a VP2 viral antigen of infectious bursal disease virus (IBDV)—a highly immunosuppressive disease in chickens—as a bivalent vaccine [49]. The rLaSota/VP2 vaccine provided 90% protection against a challenge with highly virulent AOaV-1 strain or virulent IBDV, while booster immunization induced higher levels of antibody responses against both AOaV-1 and IBDV, as well as full protection against both viruses. 

#### 4.1.4. Infectious Laryngotracheitis Virus [ILTV]

Infectious laryngotracheitis is a major disease of the respiratory tract in chickens caused by the herpes virus—infectious laryngotracheitis virus (ILTV] [65]. In order to improve the safety of the current ILTV attenuated live vaccines, ILTV vaccines based on AOaV-1-vector were developed [52,53], inducing a high level of neutralising antibodies and fully protecting chickens against the challenge with virulent ILTV and AOaV-1. These results show that recombinant AOaV-1 can be used for other avian pathogens as a vaccine vector.

### 4.2. Recombinant AOaV-1 Based Vaccines in Animals

Recombinant AOaV-1 have been utilized in the development of vaccines for use in cattle, sheep, dogs, cats, pigs, horses, and camels. These recombinant vaccines were developed for protection against viral pathogens such as Middle East Respiratory Syndrome Corona Virus (MERS-COV) [66], Bovine Herpesvirus 1 (BHV-1) [67], Bovine Ephemeral Fever Virus (BEFV) [68], Rift Valley Fever Virus [69], Vesicular Stomatitis Virus (VSV) [70], Canine Distemper Virus (CDV) [71], Rabies Virus (RV) [72], Nipah Virus (NiV) [73], and West Nile virus [74] (Table 2). Collectively, these studies showed the efficacy of AOaV-1 as a vaccine vector not only limited to poultry but can also be extended to other animals such as camel, cattle, sheep etc. Taking advantage of the non-pathogenic nature of AOaV-1 for non-avian species, further research can be directed into the development of AOaV-1 as a vaccine vector for other animal species, and, more importantly, against diseases without existing control strategies and/or facing numerous challenges.

#### 4.2.1. Middle East Respiratory Syndrome Corona Virus (MERS-COV)

Middle East respiratory syndrome corona virus (MERS-CoV) or Camel flu is a deadly respiratory disease first identified in 2012 in Saudi Arabia [75] that poses a serious public health threat. Bats and camels are known reservoir of the virus; however, due to the high frequency of camel–human contact in the Middle East than bat–human contact, the virus is more likely to be transmitted to human via camels, which were recently declared the virus’s primary reservoir [76,77,78]. At the moment, there are no approved treatment regimens or vaccines for MERS. In an effort to develop vaccines against MERS, Liu et al. [66] generated avirulent recombinant AOaV-1 LaSota strain expressing the MERS-CoV S protein and assessed its immune response in mice and Bactrian camels. The recombinant AOaV-1 expressing MERS S protein exhibited high growth titers in embryonated eggs and was not pathogenic to poultry or mice. Additionally, significant levels of MERS-CoV-specific neutralising antibodies were observed in mice, while in camels, the recombinant AOaV-1 induced long-lasting MERS-CoV specific neutralising antibodies [66]. Although the challenge study in camels was not conducted due to situational limitations, the study was able to demonstrate the potential of recombinant AOaV-1 expressing MERS-CoV S protein as a veterinary vaccine candidate against MERS-CoV infection.

**Table 2:** Candidate AOaV-1-vectored vaccines for veterinary use.

Pathogen	AOaV-1 Backbone	Antigen	Insert Site	Animal Model	Vaccination (Route)	References
MERS-CoV	La Sota	S	P/M	Mouse/Camel	i.m.	[32]
BEFV	La Sota	G	P/M	Mouse/Cattle	i.m.	[68]
BHV-1	La Sota	gD	P/M	Calf	i.n./i.t.	[67]
Rabies	La Sota	G	P/M	Mouse/Cat/Dog	i.m.	[72]
PRRSV	La Sota	GP5; GP3 + GP5	P/M	Piglet	i.m.	[79]
NiV	La Sota	G or F	P/M	Pig	i.m.	[73]
VSV	La Sota	G	P/M	Mouse	i.m.	[70]
CDV	La Sota	F or H	P/M	Mink	i.m.	[71]
WNV	La Sota	PrM and E	P/M	Mouse/Horse/Chicken	i.m.	[74]
RVFV	La Sota	Gn	P/M	Cattle	i.n./i.m.	[69]

Middle East respiratory syndrome coronavirus (MERS-CoV); bovine ephemeral fever virus (BEFV); bovine herpesvirus-1 (BHV-1); porcine reproductive and respiratory syndrome virus (PRRSV); Nipah viru (NiV); vesicular stomatitis virus (VSV); canine distemper virus (CDV); West Nile virus (WNV); Rift Valley fever virus (RVFV); spike (S); glycoprotein (G); glycoprotein D (gD); glycoprotein 3/5 (GP3/5); fusion (F); (hemagglutinin (H); E) envelop proteins; pre-membrane protein (prM); aminoterminal glycoprotein (Gn); phosphoprotein (P); matrix (M); intramuscular (i.m.); intranasal (i.n.); intratumoral (i.t.).

#### 4.2.2. Bovine Herpesvirus 1 (BoHV-1)

Infection of cattle with bovine herpesvirus 1 (BoHV-1) can lead up to upper respiratory tract disorders, conjunctivitis, or genital disorders, leading to significant economic losses [67,80]. To combat this virus, a variety of vaccines are available to protect the cattle from infection; however, these vaccines are not without flaws. A significant disadvantage of the currently available modified live BoHV-1 vaccines is their ability to induce latent infection, with the risk of subsequent reactivation [67]. In an effort to develop more effective vaccines, Khattar et al. [67] developed a recombinant AOaV-1 vaccine expressing the BoHV-1 gD protein. In calves, a single intranasal/intratracheal immunisation with the recombinant AOaV-1 vaccine (2 × 10^7^ PFU/dose) resulted in significant mucosal and systemic antibody responses. These findings indicate the potential of AOaV-1 as a vaccine vector to develop a mucosal vaccine against BHV-1 infection in cattle.

#### 4.2.3. Rift Valley Fever Virus (RVFV)

Rift Valley fever virus (RVFV) is transmitted through contaminated animal products and arthropod vectors, causing huge outbreaks in ruminants in a number of countries, and has zoonotic concerns for humans [81,82]. Live attenuated vaccine (Smithburn strain) and inactivated RVFV vaccines are frequently used to control the disease. Inactivated vaccines are safer, but less immunogenic, thus often requiring boostering or adjuvants, while the administration of live vaccines poses safety questions for young animals [8]. Kortekaas et al. [69] developed a recombinant AOaV-1 vaccine expressing the RVFV Gn and Gc glycoproteins to improve the vaccine safety [69]. Immunisation with recombinant AOaV-1-Gn + Gc vaccine (10^7.3^ TCID_50_/dose) elicited high levels of neutralising antibodies specifically for RVFV. These results show high levels of immunogenicity and highlight the potential of AOaV-1 as a vaccine vector against RVFV. 

#### 4.2.4. Canine Distemper Virus (CDV)

Canine distemper virus (CDV) infects many carnivores and is responsible for several outbreaks of high mortality [71]. Some exotic species, such as mink and ferret, cannot be safely immunised with the current CDV live vaccine. As vaccine candidates, AOaV-1 strain LaSota expressing envelope glycoproteins, H protein and F proteins, respectively, was created [71]. The recombinant AOaV-1 expressing the hemagglutinin (H) induced higher titers of CDV neutralisation antibodies in immunised minks than that expressing the fusion protein with complete protection against a virulent CDV challenge [71]. This study concluded that recombinant AOaV-1 expressing the CDV H protein is a safe and effective vaccine candidate against CDV in mink, but its efficacy in other carnivore species needs to be investigated.

#### 4.2.5. Rabies Virus (RV)

In humans and animals, rabies virus (RV) causes a fatal neurological illness [72]. The AOaV-1 strain LaSota expressing the rabies virus glycoprotein G (rL-RVG) was tested in order to develop an effective, safe, and affordable rabies vaccine. In dogs and cats, intramuscular vaccination with rL-RVG induced strong and long-lasting protective neutralising antibody responses against rabies virus [72]. Despite the fact that three vaccination doses were administered, the second dose elicited the strongest immune responses in both cats and dogs. This study showed that AOaV-1 vectored vaccine can protect dogs from rabies virus and can be used to control rabies in high-risk human individuals.

#### 4.2.6. Bovine Ephemeral Fever Virus (BEFV]

The bovine ephemeral fever virus is transmitted by arthropod, causing febrile acute disease in cattle and water buffalo, as well as causing bovine ephemeral fever (BEF). Zhang et al. [68] reported that recombinant AOaV-1 expressing the BEFV glycoprotein (G) (rL-BEFV-G) is safe in mice and cattle through induction of high levels of neutralising antibodies, and showed protection against a BEFV challenge. These results suggested the potential for AOaV-1 to be a promising candidate vaccine against BEFV [68].

#### 4.2.7. Vesicular Stomatitis Virus (VSV)

The animal industry suffers significant losses as a result of vesicular stomatitis virus (VSV). A recombinant AOaV-1 expressing the glycoprotein (G) of VSV (rL-VSV-G) was created, and its pathogenicity and immune protective efficacy in mice were assessed [68]. It was concluded that recombinant rL-VSV-G induced a high titer of neutralising antibodies against VSV with a lower viral load in mouse organs after challenge [68]. These findings suggest that rL-VSV-G could be a promising candidate vaccine against vesicular stomatitis (VS).

#### 4.2.8. West Nile Virus (WVN)

The West Nile Virus (WNV) is an emerging zoonotic disease that is harmful for human and animal health [83]. A flexible vaccine, which can be administred through different routes for different species, is essential for effective vaccination of susceptible hosts to protect against WNV and significantly reduce the viral transmission between animals and humans [74]. Wang et al. have developed and assessed the immunogenicity of recombinant avirulent AOaV-1 LaSota strain expressing pre-membrane-E (PrM/E) WNV proteins (designated rLa-WNV-PrM/E) in mice, horses, ducks, and geese, which induces significant levels of WNV-neutralizing antibodies, CD4+, and CD8+ T cell responses upon delivery via intramuscular immunization, oral or intranasal immunization, respectively. These results support the use of rLa-WNV-PrM/E as a promising vaccine candidate against WNV [74].

## 5. Current Application of AOaV-1 as Vaccine Vector for Emerging and Remerging Human Viruses

Conventional vaccines have effectively reduced the burden for many infectious disease, i.e., small pox eradication and substantially controlling diseases such as polio, tetanus, diphtheria, and measles [84]. Live attenuated viruses, inactivated viruses, or recombinant subunit-based vaccines are traditional platforms for the development of vaccines, which often contribute to a long-term immunity of many infectious human and/or animal viruses. However, a majority of them are not suitable for human use due to safety concerns, poor efficacy, or easy impracticability, and are not always suitable or even feasible in outbreak situations [84]. Additionally, scenarios of outbreaks may limit the development and or productivity of conventional vaccines.

A number of challenges need to be overcome to prove the efficacy of these conventional vaccines in the face of an emerging or future pandemic [84]. One of the key problems for pandemic preparedness is the unpredictable nature of emerging pathogens and zoonosis that poses a permanent threat to the population, as with SARS-CoV-2. SARS-CoV-2 outbreaks revealed the possibility of the known pathogens for mutations and adaptation to a new host or environment with impermissible consequences for their immunogenic properties and the seriousness of the symptoms they produce. The risk of such events is high in RNA viruses, whose high mutation rates favour adaptability, as demonstrated by recent epidemics and pandemics.

Since the goals of the vaccine remain undefined before an outbreak, time remains an important obstacle to effective development of the vaccine. The average development time currently exceeds 10 years for conventional preclinical vaccines [85], highlighting the dire need for new approaches which permit extremely rapid development and licencing to prevent the emerging outbreak from spreading worldwide.

Another major problem is the cost of vaccine development and production; the development of a new vaccine candidate with established technologies is estimated to exceed USD 500 million with additional costs for setting up equipment and facilities between USD 50 million and 700 million [86]. Although some vaccine development costs cannot be avoided to meet the necessary safety standards, validation and production costs are high in every vaccine requirement for dedicated manufacturing processes and facilities in most conventional vaccine technologies. In addition, new technologies are required in order to support more cost-efficient vaccination production, particularly in light of resource limited environments and the fact that emergencies represent niche markets. The second problem is the manufacturing capacity of established methods, often inadequate for global vaccination. Although the potential threat is recognised, vaccine production technology, such as the COVID-19 vaccine, is still problematic in its production capacity to meet peak demands of a disease. An example is that the potential influenza pandemic vaccine production capacity could theatrically support vaccination of 43% of the population with two doses of the vaccine in 2015 through WHO efforts [87].

In 2015, only 5% of influenza vaccine doses were distributed to South-east Asia, the Eastern Mediterranean, and Africa in the WHO regions, which represent about half of the world’s population, but the distribution of vaccine products worldwide is far from equal between the developing and industrialised countries [88]. Moreover, most currently authorised vaccines would require 3–5 months from virus identification to the distribution of vaccines, providing the virus with ample time to spread globally. Therefore, in the event of pandemic risks, technology that allows quick manufacturing of a large number of vaccines is absolutely necessary.

The use of recombinant vectors as a vaccination tool for human pathogens was therefore critical, due to their ability to express high-level foreign proteins in host cells, which leads to a strong, long-term immune response to the target protein. As AOaV-1 is an avian paramyxovirus, a major advantage of the vaccine platform is that the issue of anti-vector pre-existing immunity is not considered a major factor. Further, several AOaV-1 strains are licensed and readily available for use as veterinary vaccines. Although even small exogenous transgenes may significantly lower the yield of recombinant viruses, most recombinant AOaV-1 vectors can be propagated to high titers in chicken eggs and even some cell lines [7].

Recombinant AOaV-1 has also been used as a viral vector in the delivery of vaccine antigen for humans [89]. Currently, AOaV-1 vaccine candidates both respiratory and non-respiratory diseases include SARS-CoV-2 [14,90], SARS-CoV [91], EBOV [92], HIV-1 [15,30], HPAIV H5N1 [22], RSV [93], and HPIV-3 [18] (Table 3). 

### 5.1. Severe Acute Respiratory Syndrome Coronavirus 2 (SARS-CoV 2)

The pandemic caused by the Severe Acute Respiratory Syndrome Coronavirus 2 (SARS-CoV 2) has already infected millions and claimed the lives of many, causing unprecedented economic losses globally [99]. At the moment, a safe and effective vaccine is critical to control the current pandemic and prepare for future pandemics. Due to the urgency of developing an effective vaccine, various platforms (i.e., mRNA vaccines, inactivated whole virus vaccines, subunit vaccines, DNA vaccines, and viral vector vaccines) are being utilised [100]. One of the candidate vaccines developed against SARS-CoV-2 is the recombinant AOaV-1 expressing SARS-CoV-2 spike (S) protein. We recently engineered AOaV-1 expressing SARS-CoV-2 S protein, wherein results showed robust growth in embryonated chicken eggs and successful expression of the S protein in infected cells [14]. Sun et al. [90] have reported that mice immunised twice (10 ug and 50 ug/each) intramuscularly have developed high levels of spike-specific neutralizing antibodies and exhibited increased protection against SARS CoV-2 challenge [89]. AOaV-1 possesses characteristics that merit consideration in terms of vaccine safety and efficacy [14]. Due to the presence of an abundance of excellent data on the safety of AOaV-1 in humans, the development of an AOaV-1 vectored vaccine may result in an efficacious countermeasure for controlling the current SARS-CoV-2 pandemic and any future pandemics [101].

### 5.2. Severe Acute Respiratory Syndrome-Associated Coronavirus (SARS-CoV)

AOaV-1 was evaluated as a vector for severe acute respiratory syndrome associated Coronavirus (SARS-CoV), an important emerging pathogens [91]. DiNapoli et al. [91] demonstrated that AOaV-1 is a vaccine vector for SARS-CoV with higher safety and protective efficacy. Two AOaV-1 vectors have been built in which the cleavage sequence of F protein was modified to that of the strain BC [AOaV-1-BC] and LaSota lentogenic strain (AOaV-1/VF) to express the major protective antigen, SARS-CoV spike (S) glycoprotein [91]. Two immunization doses for African Green Monkeys immunisation resulted in a robust neutralising antibody response, leading to a reduction in virus release after challenge with SARS-CoV.

### 5.3. Influenza Viruses

The potential usage of recombinant AOaV-1 strain B1 as an effective human vaccine vector was first evaluated with influenza virus (A/WSN/33) hemagglutinin (HA) protein [16]. HA protein was expressed in virions and has been cleaved, showing that the HA protein was accessible to proteolytic enzymes. Intravenous administration of mice led to higher antibody titers for the influenza virus HA protein compared to the intraperitoneal route. In addition, intravenous vaccination of mice showed that they were fully protected against a lethal dose of influenza virus, proving that AOaV-1 can be a safe vaccine vector for possible application in mammalian and avian species.

### 5.4. Ebola Virus (EBOV) 

In humans, Ebola Virus (EBOV) causes severe hemorrhagic fever with a fatality rate up to 88% [102]. Different viral vectors were used for EBOV vaccine due to the limitations of inactivated vaccines. The recombinant LaSota strain of AOaV-1, expressing the envelope EBOV GP, has been developed to overcome the high seroprevalence of vectors based on common human pathogens in order to evaluate its potential as an EBO vaccine [101]. 

### 5.5. Nipah Virus (NiV)

Nipah virus (NiV) is a deadly zoonotic disease that leads to human and pig fatal encephalitis (PFE) [73]. Two of the major NiV glycoproteins on the surface stimulating protective immune responses are glycoprotein (G) and fusion protein (F). The AOaV-1 strain LaSota expressing NiV-G and F proteins were evaluated for its immunogenicity in mice and pigs and showed its potential to protect animals from NiV infections [73]. 

### 5.6. Norovirus (NoV)

The most common cause of viral gastroenteritis in humans of all ages is Norovirus (NoV) [103]. In the cell culture system, NoV’s inability to grow has greatly impeded the development of effective vaccine, while baculovirus expression system virus-like particles (VLPs) were frequently used as candidates for NoV vaccines to overcome this obstacle. The capsid protein ORF2 (VP1) of the NoV strain VA387 (GII.4) was expressed by modified Beaudette C (BC) strain through changing its multibasic cleavage site sequence of the F protein and the HN protein to that of LaSota strain [104]. This study showed that AOaV-1 is highly likely to develop a live vaccine-based candidate for NoV by expression of the VP1 protein on the surface of modified BC strain. Alternatively, large quantities of recombinant viruses produced in allantoic fluid of embroynted eggs or in cell culture medium can be a cost-effective method to manufacture a VLP-based vaccine, which is a base to develop rAOaV-1-vectored vaccines for other non-cultivable pathogens of humans. In addition, rAOaV-1-vectored vaccine induced higher levels of serum, cellular, and mucosal immune responses than those induced by baculovirus-expressed VLPs, suggesting that r AOaV-1 is an efficient system for delivering the VP1 protein in vivo and can be a good candidate as a live-attenuated vaccine against NoV infection.

### 5.7. Respiratory Syncytial Virus (RSV)

In infants and elderly people, Respiratory Syncytial Virus (RSV) is a major cause of severe lower respiratory tract disease, so there is a high demand to develop an effective RSV vaccine [105,106]. Due to the ability of AOaV-1 to induce a strong Interferon (IFN)- α/β response, it was chosen as a viral vector, and AOaV-1 strain B1 was employed to express RSV fusion (F) glycoprotein in order to develop an RSV vaccine [92]. In addition, this study also emphasises the adjuvant effect for potent IFN induction mediated AOaV-1 vector. The RSV F protein was more immunogenic than live RSV when presented with the AOaV-1-F, correlating with an increased AOaV-1 capacity for activating the antigen-presenting cells and inducing high levels of IFN-α/β, leading to protection from RSV challenge [93].

### 5.8. Human Immunodeficiency Virus (HIV)

A recombinant AOaV-1 expressing simian immunodeficiency virus (SIV) gag protein was tested to evaluate the AOaV-1 vaccine potential against HIV infection that induces Gag- specific cellular immune responses in mice [107]. Intranasal administration has induced the strongest protective immune response against challenge following a booster vaccine administration, which suggests that AOaV-1 vectors may be appropriate as an HIV vaccine candidate.

Furthermore, the AOaV-1 vector was used to express the Gag and Env proteins and optimise Gag’s expression levels with different insertion sites in the AOaV-1 genome [6,15,30,107,108]. It was found that the insertion of the codon-optimized Gag between AOaV-1’s P and M genes resulted in high protein expression and an improved immune response to HIV in mice [94]. Another study showed induction for high systemic and mucosal antibodies to gp160 Env protein in guinea pigs through AOaV-1 vector [30]. In mice, this method was effective to induce cellular and protective immune responses for HIV-1 Env and Gag against challenge with vaccinia viruses, which indicates that vaccination with a single AOaV-1 vector co-expressing Env, and Gag is a promising strategy for enhancing the immunogenicity and protective efficacy against HIV [30].

## 6. Concluding Remarks

AOaV-1 is an attractive vaccine vector with a proven record of safety and effectiveness for live attenuated vectored vaccines against respiratory viruses; however, further research is needed to improve the quality and effectiveness of AOaV-1 vaccine platforms for both human and animal pathogens. Despite the fact that the AOaV-1 vector is seen to be a promising vehicle, there has been some dispute concerning its safety, i.e., the possibility of recombination between AOaV-1 vaccine strains and wild- type (wt) strains, as well as between AOaV-1 and the host [109,110,111]. Collins et al. [112] have stated that recombination is a rare occurrence, and that genetic exchange is not a practical problem in vaccinology. Furthermore, insertion of foreign genes reduces the virus’s virulence and tropism rather than increasing it. More crucially, Song et al. have revealed that AOaV-1 recombination is not as widespread as thought, and that the recombination that was discovered was most likely artificial, coming from sample contamination and sequencing errors and therefore proposal to safe administration of vaccines is warranted [113,114,115,116,117].

Importantly, AOaV-1 vectors induce not only strong humoral and cellular responses but also mucosal immune reactions. Thus, for mucosal vaccines, AOaV-1 can be a vector of choice. These platforms are equally applicable for animal vaccines due to the ability of AOaV-1 to infect a wide spectrum of non-avian species. In addition, AOaV-1 is a promising vector for humans, as most individuals do not exhibit pre-existing immunity to AOaV-1. Finally, AOaV-1 as a viral vector can be extremely helpful in presenting naturally developed viral antigens to the immune system. In general, viral vectors are more immunogenic than inactivated/killed virus vaccines, have a better safety profile than many live attenuated virus vaccines, and can express higher levels of foreign genes in vivo for longer period of time. Furthermore, viral vectored vaccines present the desired antigens to the immune system in their normal, correct conformation, a method that recombinant subunit vaccines struggle to accomplish. 

## Figures and Tables

**Table 3:** Candidate AOaV-1-vectored vaccines for human use.

Pathogen	AOaV-1 Backbone	Antigen	Insert Site	Animal Model	Vaccination (Route)	References
HIV-1	Hitchner B1	Gag	P/M	Mouse	i.n.	[15]
HIV-1	La Sota	Gag	P/M	Mouse	i.n.	[94]
HIV-1	La Sota	Gag; Env; Gag + Env	Env- P/M and Gag- HN/L; Gag- O/M and Env- HN/L; Env + Gag- P/M; Env- P/M; Gag- P/M	Guinea pigs/Mouse	i.n.	[30]
SIV	La Sota	gp160	P/M	Guinea pigs/Mouse	i.n.	[95]
EBOV	Beaudette C and La Sota	GP	P/M	Rhesus monkeys	i.n/i.t.	[92]
EBOV	Chimeric AOaV-1	GP	N/P, P/M, and M/F	Guinea pigs	i.n.	[96]
HPIV-3	Beaudette C and La Sota	HN	P/M	African green monkeys and rhesus monkeys	i.n./i.t.	[18]
RSV	Hitchner B1	F	P/M	Mouse	i.n.	[93]
Poliovirus	La Sota	P1 and 3CD	P1- P/M and 3CD- HN/L	Guinea pigs	i.n.	[97]
Lyme	LaSota/VF	BmpA + OspC	P/M	Hamsters	i.n./i.m./i.p.	[98]

Human immunodeficiency virus-1 [HIV-1]; simian immunodeficiency virus (SIV); Ebola virus (EBOV); human parainfluenza virus type-3 (HPIV-3); respiratory syncytial virus (RSV); group specific antigen (Gag); envelope glycoprotein gp160 (Env); envelope protein gp160 (gp160); (GP); hemagglutinin (HN); fusion (F); capsid protein precursor (P1); viral protease (3CD); basic membrane protein A (BmpA); outer surface protein C (OspC); phosphoprotein (P); matrix (M); large polymerase (L); nucleocapsid (N); intranasal (i.n.); intratumoral (i.t.); intraperitoneal (i.p.); intramuscular (i.m.).
